# Human-scale tissues with patterned vascular networks by additive manufacturing of sacrificial sugar-protein composites

**DOI:** 10.1016/j.actbio.2020.06.012

**Published:** 2020-09-01

**Authors:** Hoda M. Eltaher, Fatima E. Abukunna, Laura Ruiz-Cantu, Zack Stone, Jing Yang, James E. Dixon

**Affiliations:** a Regenerative Medicine & Cellular Therapies Division, School of Pharmacy, The University of Nottingham Biodiscovery Institute (BDI), University of Nottingham, Nottingham, NG7 2RD, UK; b Protowax Ltd., 150 Ashley Road, Hale, Altrincham, Cheshire, WA15 9SA, UK; c Department of Pharmaceutics, Faculty of Pharmacy, Alexandria University, 21521, Egypt

**Keywords:** Vascularization, 3D printing, Tissue engineering, Sugar-protein composite

## Abstract

Combating necrosis, by supplying nutrients and removing waste, presents the major challenge for engineering large three-dimensional (3D) tissues. Previous elegant work used 3D printing with carbohydrate glass as a cytocompatible sacrificial template to create complex engineered tissues with vascular networks (Miller et al. 2012, Nature Materials). The fragile nature of this material compounded with the technical complexity needed to create high-resolution structures led us to create a flexible sugar-protein composite, termed Gelatin-sucrose matrix (GSM), to achieve a more robust and applicable material. Here we developed a low-range (25–37˚C) temperature sensitive formulation that can be moulded with micron-resolution features or cast during 3D printing to produce complex flexible filament networks forming sacrificial vessels. Using the temperature-sensitivity, we could control filament degeneration meaning GSM can be used with a variety of matrices and crosslinking strategies. Furthermore by incorporation of biocompatible crosslinkers into GSM directly, we could create thin endothelialized vessel walls and generate patterned tissues containing multiple matrices and cell-types. We also demonstrated that perfused vascular channels sustain metabolic function of a variety of cell-types including primary human cells. Importantly, we were able to construct vascularized human noses which otherwise would have been necrotic. Our material can now be exploited to create human-scale tissues for regenerative medicine applications.

**Statement of Significance:**

Authentic and engineered tissues have demands for mass transport, exchanging nutrients and oxygen, and therefore require vascularization to retain viability and inhibit necrosis. Basic vascular networks must be included within engineered tissues intrinsically. Yet, this has been unachievable in physiologically-sized constructs with tissue-like cell densities until recently. Sacrificial moulding is an alternative in which networks of rigid lattices of filaments are created to prevent subsequent matrix ingress. Our study describes a biocompatible sacrificial sugar-protein formulation; GSM, made from mixtures of inexpensive and readily available bio-grade materials. GSM can be cast/moulded or bioprinted as sacrificial filaments that can rapidly dissolve in an aqueous environment temperature-sensitively. GSM material can be used to engineer viable and vascularized human-scale tissues for regenerative medicine applications.

## Graphical abstract

**Figure:**
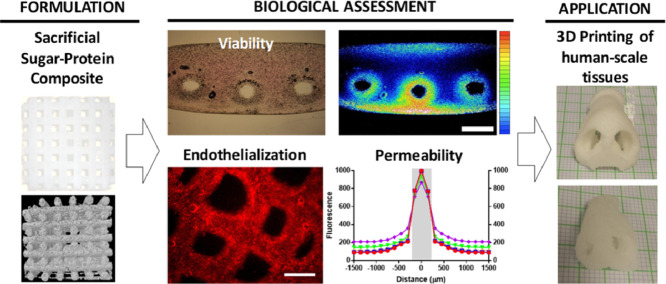

## 1 Introduction

Native tissues have high demands for mass transport exchanging nutrients and oxygen for metabolic waste [1,2]. As tissue develops these requirements are met primarily by blood perfusion through large multi-centimetre to multi-micron scale vessel networks [3]. All tissues and organs require some vascularization and this developmental angiogenic process was critical to the evolution of higher organisms [4]. When fabricating tissue for regenerative medicine applications this network must be included intrinsically within the tissue matrix and even basic vascular networks in simple physiologically-sized constructs with tissue-like cell densities have been unachievable until recently [5].

Various approaches have been developed to create perfusable vascularized tissues including layer-by-layer assembly [6], [7], [8] and recently complex additive manufacturing/3D printing/Bio-printing technologies [1,^2^,9]. Layer-by-layer technologies (which moulds a channel into one layer such that bonding of the second flat layer creates a vessel [7]) is an iterative process and can create thick, multi-layered tissues. However fabrication is slow and creates layer-to-layer seams and other structural artefacts [9]. There are also considerable design constraints on materials, channel design and cells during assembly [10]. Bioprinting using cells, matrix or both which are deposited drop-wise [9] is also relatively slow, and a serial additive process with limitations on resolution. Furthermore both technologies require direct addition of cells to the manufacturing process from the initial printed [11] or bonded layer [9] meaning that conditions need to be controlled to maintain environmental biocompatibility such as temperature, pH, humidity, nutrients and sterility [3].

Sacrificial moulding is an alternative approach in which channel networks are fabricated by creating rigid lattices of filaments which are used to prevent subsequent matrix ingress. These structures are cast inside a matrix containing cells, and post-casting removal of this material reveals a microfluidic vasculature in the bulk matrix. This work has been previously inhibited by the use of cytotoxic organic solvents, or harsh environmental conditions to create or remove the sacrificial filaments, or when casting/crosslinking the bulk surrounding matrix [6,12]. An elegant report demonstrated that this approach could be achieved using aqueous-based materials and protein-based biological matrices for the bulk material and can be used with living cells [5]. This technology used carbohydrate-dextran glass 3D printing to create a sugar sacrificial lattice. In another recent study, more designs with smaller diameter channels and smoother in-plane junctions were achieved [13]. The approach, though requiring much optimization to achieve the resolution and accuracy during fabrication, allowed perfused vascular channels to be created in thick constructs and maintained metabolic function of cells in the engineered tissue [5].

Here we describe a biocompatible sacrificial material, called GSM, which has properties beyond that of carbohydrate-dextran glass. A simple sugar-protein formulation made from mixtures of inexpensive and readily available bio-grade materials can be used to cast/mould or bio-print structures as sacrificial filaments in order to facilitate fabrication of 3D vascular networks in engineered tissues. We created a material with sufficient mechanical stiffness to support its own weight in the architecture designed and also the ability to rapidly dissolve in an aqueous environment temperature-sensitively. These properties mean that GSM is biocompatible with cells and allows the use of any synthetic or natural matrix or crosslinking strategy. Importantly GSM material can now be used to engineer human-scale tissues with functioning vascular networks.

## 2 Materials and methods

### 2.1 Materials & cell maintenance

Materials were purchased from Sigma-Aldrich (Dorset, UK) unless stated otherwise. NIH3T3 mouse embryonic fibroblasts (NIH3T3s), human umbilical vein endothelial cells (HUVECs), BJ6 fibroblast cells and HepG2 cells were obtained from the LGC Standards (Middlesex, UK). HUVEC media was supplied by Promocell (Heidelberg, Germany). immortalized human mesenchymal stem cells (iHMSCs) were created in-house as described previously [14]. DMEM media and rat tail collagen I were obtained from Invitrogen Life Technologies (Paisley, UK). Lentiviral labelling of cell lines was performed as described previously [15]. Cells were infected with enhanced green fluorescent protein (GFP)- or monomeric red fluorescent protein (mRFP)-labelled lentivirus at confluence and selected with puromycin for 7 days which produced >95% labelled cells confirmed by flow cytometry. Cells were cultured in isolation at 37°C in a 5% CO_2_ incubator. NIH3T3, HEK293T, HepG2, iHMSCs and BJ6 cells were maintained in DMEM media containing 10% (*v/v*) FCS and 1% penicillin/streptomycin. HUVEC cells were maintained in endothelial cell growth media supplemented with 0.02% (v/v) FCS, 0.004% (v/v) endothelial cell growth supplement, 0.1ng/ml epidermal growth factor, 1ng/ml basic fibroblast growth factor, 90 µg/ml heparin, 4 µg/ml hydrocortisone, and 1% penicillin/streptomycin.

### 2.2 ESM and GSM preparation

Commercially available SugarVeil™ (SugarVeil™ Products Corporation) or biological grade reformulated ESM [Sucrose (Silksugar™, British Sugar); 43.7% w/v, Egg white protein (EWP; Sigma E0500); 4.85% w/v, Starch (Miragel™ 463); 0.26% w/v, Xanthan Gum (Sigma G1253); 0.26% w/v, Maltodextrin (Sigma 419699); 0.87% w/v, final concentrations] was reconstituted according to commercial product patent details using dH_2_O [16]. GSM was formulated replacing EWP with the same concentration (48.5mg/ml) porcine skin gelatin (Sigma G1890). GSM required incubating overnight at 60˚C to complete dissolve the gelatin was placed at 37˚C prior to casting or printing.

### 2.3 Matrices and cell encapsulation

Gelatin methacrylate (GelMA) was synthesized as previously described [17]. GelMA (20% w/v final concentration) was dissolved in PBS containing photoinitiator dissolved at 60˚C (Irgacure 2959; 0.05% w/v final concentration). Cells of interest (0.1-5 × 10^7^/ml) were mixed in minimal volume with the GelMA solution and dispensed into 4 × 12mm moulds (500 µl total volume) with or without GSM structures. GelMA structures were photopolymerized (Omnicure 2000, 320-500 nm) at 200mW/cm^2^ for 30 s. Fibrin gels (20mg/ml) were created by addition of thrombin (200U/ml) to fibrinogen (20mg/ml) in PBS containing the cell suspension and dispensing around the GSM structure. Matrigel™ gels were formed by mixing loose cell pellets with Matrigel™ on ice, dispensing around the GSM structure, followed by 20min incubation at 37˚C. Alginate gels (2% w/v) were prepared using a slow setting formulation previously described [18]. Agarose gels (2% w/v) were formed by mixing ultra-low-gelling temperature agarose (Type IX, Sigma A5030, at 40˚C) with loose cell pellets and placing for 10min at 4˚C. Collagen type-I gels (2mg/ml, rat tail tip, BD-biosciences) were formed as previously described requiring 30min at 37˚C for complete gelation [18]. After crosslinking all hydrogels were placed in complete cell media (for each respective cell type) ensuring that open vessel faces were exposed to the media and placed at 37˚C. After 15 min, the culture media was replaced, and constructs were cultured under standard conditions. For perfusion, a peristaltic pump was connected to a bespoke chamber and flow rate set to 600µl/min. For 800µm constructs, these generated velocities of 2-8mm/s and a shear stress of 0.5-2 dyn/cm^2^, which are physiologically relevant values.

### 2.4 Diffusion and secretion from vasculature

Diffusion studies used FITC- and Rhodamine-labelled Dextran (4 and 70kDa, respectively, 1mg/ml) as previously described [19]. Labelled media was injected into a single engineered vessel lumen and fluorescence measured in 6 well plate format on an Infinite M200 plate reader (Tecan).

Lentiviral reporter constructs were generated as previously described [15,20]. IgH-mRFP was cloned into pSIN-PURO by PCR and HepG2 cells infected at a multiplicity of infection (MOI) of 20 and selected with puromycin for 2 weeks. IgH-mRFP release was measured by fluorometry using an Infinite M200 plate reader (Tecan). Albumin in sampled media was quantified by enzyme-linked immunosorbent assay (ELISA) using a human albumin ELISA kit (Bethyl labs). Urea in sampled media was measured by acid- and heat-catalysed condensation of urea with diacetylmonoxime to give a coloured-product that was measured spectrophotometrically (Urea Nitrogen kit; StanBio Labs).

### 2.5 PCL and GSM casting and printing

Commercially available silicon lace mats (SugarVeil™ Products Corporation) or bespoke silicon mats were used to cast/mould ESM or GSM into 2D patterns using manufacturer's guidance notes. Material was left at room temperature for >6h to achieve structurally sound matting when extracted from moulds. GSM printing used heated syringes (at 60˚C) on a 3D Discovery® bioprinter (RegenHU). PCL (M_n_ = 40,000 - 50,000 g/mol; Sigma-Aldrich, UK) was printed at 77°C using a precision extrusion deposition system as described in [21]. The material was extruded through a 23 gauge nozzle (0.33mm) steel nozzle. The printing parameters were set as: print-head travelling rate of 16 mm/s, pressure of the PCL melt chamber was 4 bar and feeder extrude rate was 20 revolutions per meter. Between PCL layers GSM was either printed (through a 21 gauge plastic tip) or applied and cast as for 2D fabrication. After fabrication PCL/ GSM composite structures were dried for >6h in a 37˚C oven, frozen at -80˚C and freeze-dried for 2 days. To remove the PCL scaffolds were then immersed in chloroform at room-temperature until all PCL was dissolved (10min-6h). Structures were washed twice in fresh chloroform and allowed to dry under laminar flow for 30min. GSM networks were either used directly with cells or were stored vacuum sealed or at 37˚C to prevent moisture absorption. For Wax moulding of GSM, we used a ProJet™ CPX 3000 plus (3DSYSTEMS) 3D printing according to manufactures instructions. Vessels to be filled with GSM were initially printed with VisiJet® S200 support material (3DSYSTEMS) which is isopropanol soluble and was removed by isopropanol bathing ready for GSM injection.

### 2.6 Construct visualization

Pre-casting images were captured by a Nikon SMZ 1500 microscope with a SPOT insight camera, or a Nikon Eclipse TS100 Microscope (Nikon Instruments, Surrey, UK). To view 3D constructs a TCS LSI super zoom confocal microscope was used (Leica Microsystems, Milton Keynes, UK).

### 2.7 Data and statistical analysis

Data are presented for cells cultured on at least three separate occasions and are expressed as mean ± SEM. Data were analysed (GraphPad Prism, San Diego CA) using unpaired T-test.

## 3 Results

### 3.1 Assessment of sugar-protein sacrificial material, ESM

Protein-sugar materials can be formed by dissolving a proteinaceous material with a thickening agent and sucrose into water. We initially investigated the use of a commercially available confectionary product (SugarVeil™; [16]) as a potential candidate as a base for material development. For translation into an experimental and biomedical context, we reformulated it using biological grade reagents. The matrix contains egg white protein (EWP; of which ovalbumin is the main constituent [22]) as the proteinaceous component and the material is created by addition of boiling (>95°C) distilled water and mixing (40 rpm for 5 min) which achieves the flowable protein-foam. We termed our reformulated material EWP-sucrose matrix (ESM). The ESM foam has a pH value of 7.2, at low density (0.4g/ml) and has sufficient viscosity (~75,000 cP) to allow low aperture printing but accuracy at high-resolution (>50µm filament diameter) [16]. The hygroscopic properties of ESM were advantageous as when dried then humidified, it possesses up to 50% elasticity meaning that it is not as brittle as simple carbohydrate glasses. This transition is reversible as at ‘room’ relative humidity (RH; 30% RH), it dries to form a hard and brittle material; however placing for 5 min at 95% RH (on a release substrate such as silicon) it softens recreating the mouldable, flexible and stretchable material.

Uniaxial compression testing of ESM in 95% RH or partially hydrated (30% RH) conditions showed elastomeric and stiff mechanics, respectively. Importantly when ESM was completely dehydrated it is significantly stronger than carbohydrate-only or dextran containing formulations [16].

### 3.2 ESM as a biocompatible sacrificial material

**Fig. 1:**
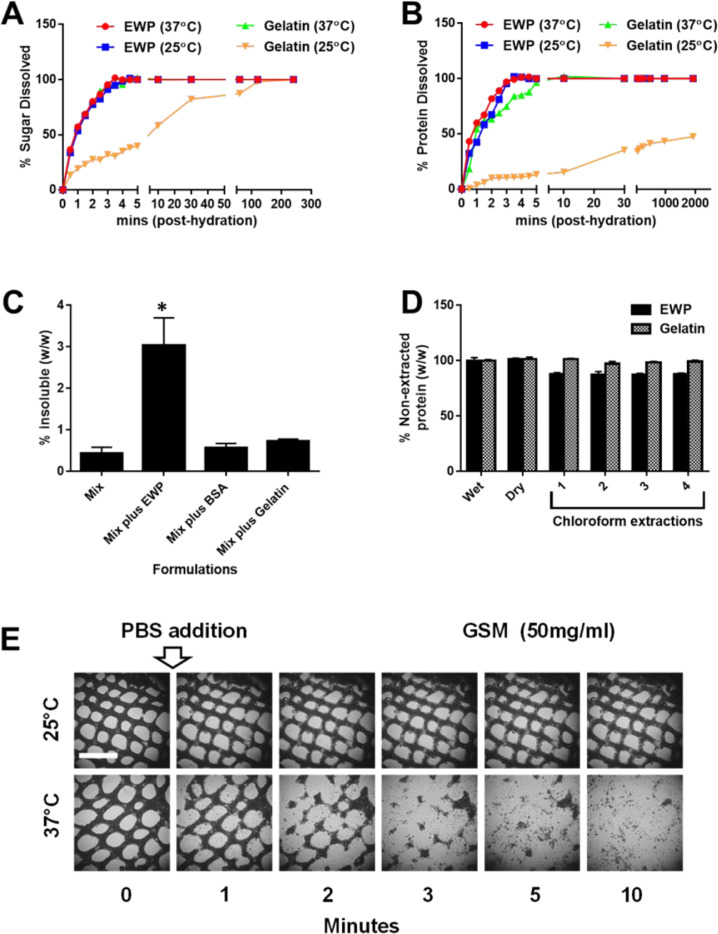
Reformulation of ESM into temperature-sensitive GSM. Fig. 1. Formulation of ESM into temperature-sensitive GSM. The reformulated ESM material using biological grade chemical was modified to contain gelatin rather than EWP to confer temperature-sensitive properties to the material, termed GSM. (**A-B)** Examination of the dissolution processes of ESM and GSM. Scientific-grade ESM and GSM formulations were tested in the same conditions as in (A) but PBS was sampled and tested for sucrose (sugar component of both materials); (**A)** and protein (EWP or gelatin components of ESM and GSM, respectively; (**B)** release using sucrose and BCA assays, respectively. Sugar dissolution was rapid (full by ~4 min.) for both materials except for GSM at 25°C which was complete after ~2 h) post-hydration with PBS. Protein dissolution was similar with all protein dissolved within 4 min (~5 min. for GSM at 37°C) except for GSM at 25°C which was never fully dissolved even after long incubations (32 h only ~50% dissolved) with the overall structure remaining intact. (**C)** Estimation of the insoluble material after full dissolution of ESM (Mix plus EWP, GSM (Mix plus Gelatin) or using BSA as the protein element (Mix with BSA; BSM). Material was incubated in excess PBS (1g material in 10ml PBS) for 48 h at 37°C, centrifuged (20,000 x g) and the pelleted material dried and compared to the original mass. EWP in ESM leaves a significant insoluble fraction (~3% w/w) whereas BSA and Gelatin leave no further insoluble material over the mix alone (~0.4% w/w). Importantly these formulations when fully dissolved could be passed through 0.4µm disc filtered demonstrating that any residual insoluble material was smaller than this pore size. (**D)** The protein content of ESM and GSM is stable even after multiple chloroform extractions. (**E)** GSM (50mg/ml) was cast by a silicone-mould screening technique into mesh pattern and tested for temperature sensitivity by addition of PBS at 25°C or 37°C. After 10 min static incubation at 37°C the mesh was fully dissolved, at 25°C the material retained its structure. Bar is 2 mm. * *p*<0.05.

For ESM to be employed sacrificially it must dissolve quickly in supersaturated conditions and diffuse away rapidly leaving its previous space completely vacant for diffusion and perfusion. We tested the dissolution kinetics of ESM in comparison to the carbohydrate-dextran blend used in a previous study [5]. We demonstrated that the half-dissolution time for ESM was 43±7 and 31±4 s at 25˚C and 37˚C, respectively, which is slower but comparable to carbohydrate-dextran kinetics (9±3 s). We noted that some material from ESM did not fully dissolve even with prolonged incubation (Fig. 1). Centrifugation (at 15k x g) revealed approximately 3.1±0.2% of ESM mass (6.2±0.4% of the dry mass) was insoluble after dissolution (Fig. 1C). We determined that the majority of insoluble mass was attributable to denatured EWP [23] as formulations lacking EWP had 0.30±0.09% insoluble mass. Next, we assessed its biocompatibility by incubating solutions of dissolved ESM with immortalized human mesenchymal stem cells (iHMSCs) and assessing viability and proliferation over a 12h culture period (Fig. S1A). This is important as the sugar-content does not osmotically damage living cells. Even at concentrations of 20% w/v (0.2g/ml), there was no significant effect on proliferation (doubling times of 21±2h at day 3 versus 19.3±1.3h for controls) nor viability (95±3 viability at day 3 versus 97±3 for controls) compared to controls.

It is important that any sacrificial material is compatible with the use of matrices crosslinked by photochemical reactions [24]. Unlike carbohydrate or carbohydrate-dextran blends, ESM is not completely optically transparent (as a constituent is starch-based and contains denatured EWP). However, its absorption properties indicated it did transmit significantly at wavelengths compatible with photopolymerization [25] including UV, visible and near IR ranges (Fig. S2).

### 3.3 Reformulation to create temperature-sensitive GSM

A major drawback of carbohydrate-dextran blends described in a previous study is its lack of stability during casting and crosslinking of the bulk matrix surrounding it [5]. The authors of that study coated fabricated lattices with poly(lactic-co-glycolic) acid (PLGA) dissolved in chloroform to improve its stability, allowing it to prevent matrix ingress sufficiently long to retain the sacrificial structure on dissolution. Slow setting hydrogels such as collagen type-I (~30 min to set) which rely on fibrillogenesis by strand precipitation with a pH change [26], are problematic with the carbohydrate-dextran system and were not tested in the previous study [5]. ESM also performs poorly if the encasing matrix is slowly crosslinking, so we reformulated the material removing EWP and replacing with bovine serum albumin (BSA; termed BSA-sucrose matrix or BSM) or porcine skin gelatin (gelatin; termed gelatin-sucrose matrix or GSM) or blends of both (Fig. 1A–C). Gelatin is a temperature responsive protein [27] which gels at sufficiently high concentrations at <32˚C. Removing EWP led to a higher density material (i.e. less foaming) and removed the majority of insoluble material remaining after dissolution (Fig. 1C, 0.28±0.07% for complete replacement with gelatin). Importantly, increasing amounts of gelatin improved the retention of volume upon superhydration (submerging in dH_2_O) at temperatures <32˚C. Concentrations >30mg/ml gelatin were sufficient to retain the structure which converted from a stiff brittle material into a soft hydrogel upon complete hydration. We continued study of the material entirely proteinated with gelatin (48.5mg/ml), Gelatin-sucrose matrix (GSM), as it retained the best temperature-responsive properties and still dissolved rapidly when placed at permissive (>32˚C) temperatures (Fig. 1A and B; 9±2 h verses 35±6 s at 25˚C and 37˚C, respectively).

We empirically determined a GSM concentration (60% w/v in dH_2_O) that yielded a final hydrated composition which flows readily through extremely small apertures (30 gauge; 159µm) when heated >32˚C and thus can be formed into detailed and complex patterns (extruded filament diameter of 100µm). GSM is more optically transparent than ESM either at 25°C or 37°C and this therefore compatible with photoactive fabrication strategies (Fig. S2).

### 3.4 Multiscale vascular networks of GSM

We initially investigated 2D fabrication of GSM patterned sheets (Fig. 1, S3). GSM can be simply extruded on a release substrate (silicon sheeting) or can be silk screen moulded [28] into complex patterned vascular sheets which can be easily dissolved (Fig. 1E, S3). Importantly the rapidity of GSM removal was retained when encased in hydrogels meaning that the high sugar concentration of GSM would have minimal effect on cell viability. The sugar element rapidly hydrated and at 37°C gelatin quickly liquefied (<1min) and flowed from vessels even in complex structures. Prior to use these sheets can be stored for several weeks and, if required, can be softened or hardened by varying humidity before further fabrication. Furthermore, these 2D patterns can be combined and bonded with addition and dehydration of more GSM to create infinite possible vessel configurations before bulk material fabrication. GSM sheets may also be rolled into vessels creating tubular structures with vascularized walls (Fig. S4). This is an interesting application which does not require bioplotting but can manually generate vascularized sheets or tubes.

Unlike carbohydrate-dextran glass [5], GSM is not completely self-supporting due to its moisture content before gelation of its gelatin component. However, when dehydrated/lyophilized, GSM is an exceptionally durable material. Therefore, for direct use in 3D fabrication we optimized the printing conditions (methods) and investigated co-fabrication materials, which allowed rapid fabrication without the need to regulate humidity of room-temperature. Many studies using thermal extrusion additive manufacturing technologies have employed polycaprolactone (PCL) as a printable biomaterial or as a scaffold for weaker or uncrosslinked materials such as hydrogel matrices [21,29]. Importantly, PCL is chloroform soluble [30], whereas all of the constituents of GSM are not (Fig. 1D). Therefore, extraction of PCL from co-engineered constructs could be achieved easily by bathing in chloroform (2–12h at room temperature). If instantaneous stronger mechanical properties were immediately required beyond that of the hydrogel used, then the PCL could be left as seen in other strategies [21]. Chloroform has been used as a method to sterilize biomass [31] and collagen hydrogels [32] for decades. Therefore this chloroform step could be used in all applications to remove bacterial and viral load of good manufacture practice (GMP) materials to generate GSM for translation. To demonstrate the approach we employed extrusion of PCL to create layer-by-layer moulds for GSM to be cast or extruded into during fabrication [21] (Fig. S5). This approach allowed the physical properties of PCL to be employed to create 3D moulds of complex architecture with GSM embedded in each layer forming the vessel structures. Between layers both PCL and GSM fused creating a continuous structure and layer intersections. After chloroform extraction of PCL the GSM structure was revealed as an inverse of the printed PCL and layers remained fused and mechanically sound (Fig. S5). We have also demonstrated this chloroform-dissolvable scaffold approach using wax-based scaffolds and a commercial printing system (ProJet™ CPX 3000 plus; 3DSYSTEMS) which can achieve structures of 10µm resolution over 50mm scales (Fig. S6). We found that fully extracted GSM structures could represent >85% accuracy when compared to the original 3D model file used for fabrication using µCT analyses.

We initially aimed to demonstrate a simple lattice structure as a sacrificial element for creating fluidic channels within monolithic cell-laden constructs. For this a cell suspension of non-crosslinked Alginate matrix is cast around the GSM construct to encapsulate the architecture. After matrix crosslinking, the temperature is increased to 37˚C to dissolve the GSM lattice and form interconnected vessels. For carbohydrate-dextran lattices [25], this process required PLGA coating of the structure in chloroform before casting of hydrogels with cells. We saw rapid release of some GSM components into the cast matrix except for the gelatin component which retained the lattice architecture during matrix crosslinking (as demonstrated in Fig. 1A & B). As we previously demonstrated for ESM, GSM does not elicit osmotic damage to cells (10% w/v leaves 95.7% viability over 24h in iHMSC cells: Fig. S1) due to the rapid removal of the material after casting. Furthermore, the temperature-sensitive GSM structure was stable in superhydrated conditions at lower temperatures (unlike carbohydrate-dextran glass) and no disruption to matrix crosslinking during dissolution was observed meaning we did not need to employ the previously described PLGA-coating approach [25]. Analyses in simple lattices of the distribution of the carbohydrate- and gelatin-constituents of GSM revealed that 78 ±11% and 95±5%, was flowed out of the formed channels rather than through the bulk construct, respectively. Our approach retained a vessel surface property that allowed unrestricted diffusion from vessels into the bulk gel (Fig. S7B and C).

To demonstrate the generic application of GSM to vessel fabrication, we patterned vascular channels with casting of cells suspended in a wide range of matrices, including those with diverse crosslinking mechanisms and slow crosslinking kinetics such as for type-I collagen (Fig. S7). The time taken for the full cell-encapsulation process is dependent entirely on the crosslinking method and GSM removal at 37˚C depending on construct architecture is complete in 10–30min. Also trapped air within hydrogels can be evident depending on the nature of the application, however this can be removed by centrifugation. We demonstrated the approaches of chain entanglement (Agarose), ionic crosslinking (Alginate), photopolymerization (Gelatin methacrylate; GelMA), enzymatic crosslinking (Fibrinogen cleaved by thrombin) and protein precipitation either completed in rapid (Matrigel™) or slow crosslinking (Rat tail Collagen Type-I). GelMA constructs crosslinked by photochemical means did not show artefacts in gelation due to shadowing; demonstrating low light absorption by the GSM (Fig. S2).

We assessed the direct effect on cell encapsulation with the GSM template and observed that iHMSC cells recovered from cast hydrogels (obtained 1 h post casting with Alginate using de-crosslinking methods from [18]) were not different from no-structure controls; demonstrating the complete process is biocompatible (Fig. S8). We repeated experiments with more sensitive cell-types and found that human induced pluripotent stem cells (HiPSCs), embryonic stem cells (HESCs), primary mesenchymal stem cells (HMSCs) and umbilical vein endothelial cells (HUVECs) can also tolerate the process with no effect on viability (Fig. S8) again suggesting no osmotic challenge to cells.

### 3.5 Cell-laden vessel wall engineering by addition of crosslinkers to GSM

**Fig. 2:**
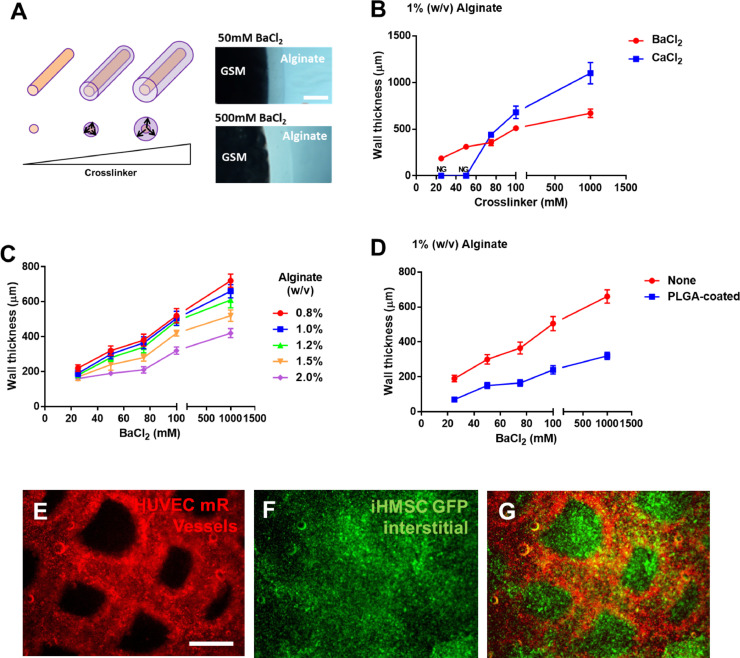
Cell loaded vessel walls and interstitial spaces using GSM. (**A)** Schematic of crosslinker diffusion from GSM to create hydrogel-coated vessel walls. Increasing amounts of crosslinker creates a thicker vessel wall upon exposure to Alginate (50mM versus 500 mM BaCl_2_). Bar is 750µm. (**B)** BaCl_2_-mediated crosslinking of Alginate can create a range of wall thicknesses (80–600µm). CaCl_2_-mediated crosslinking requires higher concentrations for crosslinking and cannot generate as thin vessel walls. (**C)** Vessel wall thickness is affected by Alginate concentration but is tuneable using BaCl_2_ crosslinking. (**D)** PLGA-coating of GSM structures before hydrogel encapsulation creates thinner vessel walls (~40µm). Vessel walls were measured by microscopy and calculating thickness using a scale calibration. (**E–G)** Vascularized constructs containing endothelial vessel walls (**E**, HUVEC-mR) and mesenchymal stem cell interstitial spaces (**F**, iHMSC-GFP) (Merged in **G**) scale bar is 500 µm.

In order to more fully recapitulate a completely vascularized tissue, the lumen provided by the sacrificial material (providing a source and sink for soluble molecules) also must be lined with mass-transport regulating endothelial cells. These cells control input and output from the lumen to the interstitial cells between vessels. Initially we proved that vessel structures were indeed linked and perfusable using laminar (rocking at 10 rpm) or pulsatile (peristaltic pump) using flow of dye-spiked media (as shown in Fig. S3). To create vessel walls lined with cells we developed two approaches; one was to seed cells under flow by resuspending in the perfusate as previously described [19]. The other approach was developed by the addition of an ionic crosslinker to the GSM formulation (which was not removed during PCL dissolution with chloroform) allowing Alginate crosslinking directly onto the GSM surface (Fig. 2) [18]. During formulation we found that addition of CaCl_2_ or BaCl_2_ crosslinkers (>1M or 100mM final concentration, respectively) stiffened the material and made the dried material more brittle and fragile mechanically. However, using tailored BaCl_2_ concentrations (10-100mM), no effect on GSM mechanical properties was observed and this allowed us to control crosslinking distance from the GSM source. Furthermore, these concentrations of BaCl_2_ for the limited cross-linking time, has no effect on cell viability (Fig. S1B). This approach has several advantages and technical implications. Directly crosslinking hydrogels to the surface of the material and tailoring the matrix (Alginate) and crosslinker formulation/concentration allowed ‘vessel walls’ to be created. These walls could be 20µm to several millimetres thick as the crosslinking distance was directly proportional to BaCl_2_ concentration diffusing from the GSM (Fig. 2A–D). Addition of cells to the matrix during crosslinking allowed endothelial cells (HUVECs) to be captured specifically in the vessel wall and allowed a secondary matrix or cell-type to be applied to form the bulk interstitial tissue (here demonstrated for collagen; Fig. 2E–G). Furthermore, as in other studies, it was possible to seed endothelial cells on the inside walls of lumen by slow perfusion if a cell adherent hydrogel was used (such as collagen). In co-cultures with iHMSC cells labelled with eGFP in the interstitial space (Fig. 2F, iHMSCs-GFP cells), endothelial cells (Fig. 2E, HUVEC-mRFP cells, labelled with mRFP1) could be fabricated first to line the original vessel structure. Importantly, we also demonstrate that the presence of endothelial cells in vessel linings slows diffusion from vessels into the interstitial scaffold creating a functional biological barrier (Fig. S9). This is evidence that the structures can be endothelialized employing our methods.

### 3.6 Metabolically dense tissues using GSM vascularization

**Fig. 3:**
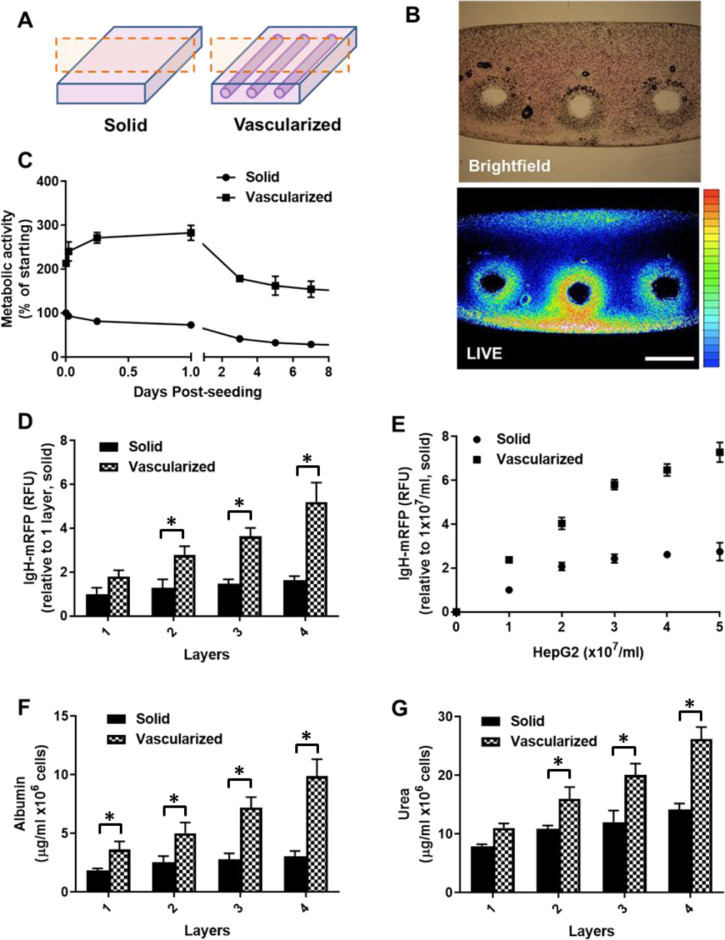
GSM-mediated vascularization promotes cell viability and functionality. **(A)** Schematic of the constructs created to test avascular versus vascularized GelMA hydrogels. (**B)** HEK293T cells (5 × 10^7^cells/ 1ml construct) were generated with and without vasculature and cultured. Constructs were sectioned at day 3 (at the plane shown in A) and stained for live cells using Calcein-AM. Brightfield and fluorescent images are shown. Metabolically active live cells appear at the media/hydrogel interfaces including those formed by vessels. Bar is 500 µm. (**C)** Constructs loaded with HEK293T cells (5 × 10^7^cells/ 1ml construct) were examined without manipulation using PrestoBlue metabolic assay over a 7 day time course. Metabolic activity was ratioed to avascular construct metabolism at time point 0 (denoted as 100%). Initial metabolic activity is higher in the vascular constructs as they promote diffusion of the assay reagent and produce a more efficient reaction condition. Vascularized constructs increase metabolism with a decrease slightly below starting levels over the 7 day period. Avascular constructs did not increase metabolism over the experiment and finished at 22.5% of the starting metabolism after the 7 days. (**D)** Constructs loaded with HepG2 hepatocytes (5 × 10^7^cells/ml) were created with different layers of vessels (in the same hydrogel volume per layer, 100µl, 1 × 10 × 10mm; depth x width x length, respectively). We used IgH-tagged mRFP expressing HepG2 cells which secrete red fluorescence into the culture media and therefore can be used to non-invasively assess cell metabolism and mimic growth factor secretion. The more vessels the more significant the IgH-mRFP secretion, increasing layers of non-vascularized hydrogel did not significant increase the amounts of IgH-mRFP produced (assayed at day 3 post-fabrication) (*n*=3). IgH-mRFP relative fluorescence units (RFU) are normalized to solid, one layer samples. (**E)** Constructs were created with different quantities of cells (0-5 × 10^7^cells/ml, with each construct layer requiring 100µl volume) with or without vascular structures. Vascularized constructs shown significantly more IgH-mRFP secreted at all densities over non-vascularized. Also vascularized constructs were able to support enhanced secretion at high cell densities than non-vascularized constructs (*n*=3). IgH-mRFP relative fluorescence units (RFU) are normalized to solid, 1 × 10^7^ HepG2 cell samples. (**F,G)** The sample constructs as described in (**D)** were assessed for secretion of **F)** Albumin and (**G)** Urea at day 3 (post-fabrication). Vascularized constructs with increased numbers of layers of vascular structures produced more albumin and urea per cell than if non-vascularized (*n*=3). * *p*<0.05.

The major goal of a vascularization technology is to facilitate the generation of dense cell-populated engineered matrices without generating necrotic areas due to poor mass transport. Therefore, cellular function must be preserved when the conditions are metabolically restrictive as seen in physiologically dense tissues. Most physiological tissues have high cell densities, such as human liver being ~6.5-18.5 × 10^7^ cells/ gram of tissue [33]. Therefore we assessed the ability of our system to support cell biology at similar initial densities (up to 5 × 10^7^ cells/ml hydrogel). We used GSM vascular sheets to assess construct metabolism as this allowed a simple visualization of the cells within constructs (Fig. 3). We loaded HEK293T cells (5 × 10^7^/ml) within GelMA matrices, perfused them with pulsatile flow and were able to show that after several days culture (up to day 3) that cells were uniformly distributed but were only metabolically active close to the gel surface by Calcein-AM staining positivity (Fig. 3A and B). Importantly we sectioned gels before staining to allow more uniform penetration of the labelling. GSM-produced vessels showed increased metabolism in the core which was highly localized around the vessel and at media exposed interfaces. Without perfusion neither control nor vascularized matrices had metabolic activity beyond the gel perimeter.

We employed two non-invasive assays for assessing longer-term metabolism [24] within the entire engineered constructs. These employed either colorimetric assays based on resazurin reduction (PrestoBlue®; Fig. 3C) over 7 days culture of HEK293T cells (5 × 10^7^/ml) or analysed secreted IgH export signal-mRFP fluorescent reporter directly from HepG2 hepatocytes (5 × 10^7^/ml) (Fig. 3D and E) [34]. Importantly we showed that, in densely populated gels, vascularization maintained cellular metabolism (Fig. 3C) and cellular secretion was enhanced (Fig. 3D and E). Although these experiments do not account for diffusion of resazurin, as penetration of reagent or release of IgH-mRFP that is directly controlled by construct porosity as well as cell metabolism, we were able to show that at high cell densities of two cell lines (which go beyond the metabolism sustained by simple diffusion) that constructs could overcome diffusion limits by addition of vessels, and this is further promoted by perfusion (Fig. 3E). We also assessed Albumin and Urea secretion (day 3 post-fabrication) from HepG2-laden constructs fabricated with different densities of vascularization (incorporating different numbers of vascular layers). These analyses showed that vascularized constructs with increased vessel layer densities produced more Albumin and Urea per cell than if non-vascularized (Fig. 3F and G, respectively).

Ideally neovascularization (or anastomosis) would be supported if the vessels remained stable and could be remodelled over time [35]. It was clear that vessels retained integrity throughout the 7 day culture period by macroscopic analyses. These data demonstrate that cell metabolism and protein secretion is supported by GSM vasculature. Cell densities at or near to physiological values can be achieved by this vasculature and subsequent perfusion illustrating the value of GSM for engineering tissues.

### 3.7 Human-scale vascularized tissue constructed using GSM

**Fig. 4:**
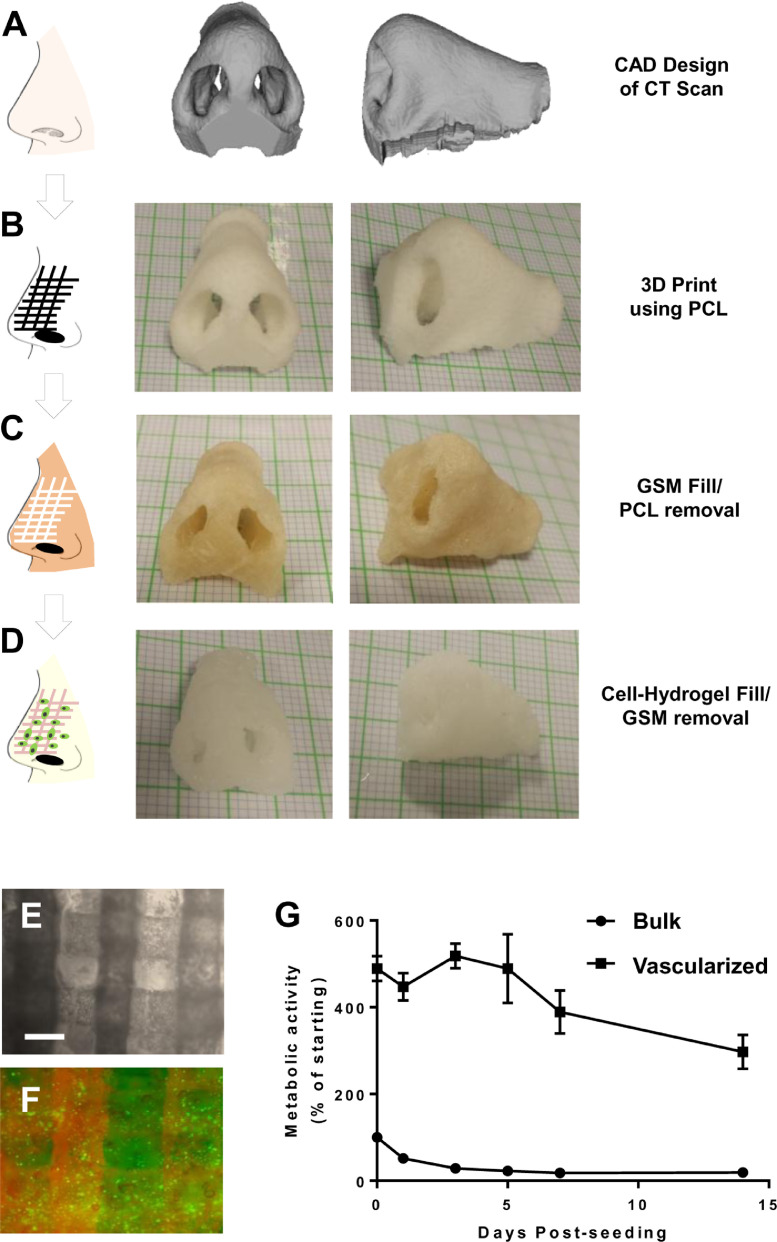
GSM-mediated vascularization of a human-scale nose constructs. **(A)** Computer-aided design projection created from a CT scan of a human nose. (**B)** 3D printed porous nose using PCL as an initial scaffold structure. Graph paper is 1mm^2^/square. (**C)** GSM filled nose with PCL removed using chloroform solubilization. (**D)** Alginate filled nose with GSM removed by incubation in phenol-red free growth media at 37°C for 3 h (120rpm mixing). GSM and Alginate were injected into the PCL and GSM structures, respectively using an air driven syringe. (**E,F)** Microscopy of a section of the nose Alginate constructs (containing 1 × 10^7^/ml iHMSC-GFP cells) showing (**E)** light microscopy of the vessels created by the sacrificial GSM, and **(F)** vessels shown by injection of TMR (fluorescent red) label and interstitial Alginate hydrogel containing iHMSC-GFP cells (fluorescent green) signal. Bar is 500 µm. (**G)** Equivalent non-vascularized and GSM-vascularized nose constructs were examined without manipulation using PrestoBlue metabolic assay over a 14-day time course. Metabolic activity was ratioed to avascular nose construct metabolism at time point 0 (denoted as 100%). Initial metabolic activity is higher in the vascular constructs as they promote diffusion of the assay reagent and produce a more efficient reaction condition. Vascularized noses increase metabolism with a decrease slightly below starting levels over the 7 day period. Avascular constructs did not increase metabolism over the experiment (*n*=3).

To fully demonstrate the utility of the system, we 3D printed a channel-containing human nose (Fig. 4) derived from a CT scan of a patient (Fig. 4A). We infilled the PCL pores (Fig. 4B) with GSM and removed the PCL by chloroform (Fig. 4C). The structure remained intact and mirrored the initial PCL scaffold and CT scan. The structure was then infilled with Alginate (1% w/v) containing iHMSC-GFP cells (1 × 10^7^/ml) yielding a vascularized and cellularized human nose construct (Fig. 4D). The vessels could be perfused with fluorescent dye TMR (By injecting into the centre of the scaffold with a syringe) and the cells imaged within the construct (Fig. 4E and F). To examine viability, we cultured constructs in rotating culture (20rpm) and compared the survival by metabolism of the nose construct compared to the same shape and volume construct without vessels (by injection moulding of Alginate). The vascularized construct possessed higher initial metabolism and retained obvious activity over a 14 day period in a rotating culture, whereas the bulk nose rapidly lost cell metabolism and retained only minimal viability at the construct surface (Fig. 4G). Vascularized noses increased in cell metabolism with a decrease slightly below starting levels over a 7 day period which is likely to be improved by using alternative hydrogels to Alginate and also be a function of the proliferation status and not simply viability. Avascular constructs did not increase metabolism whatsoever highlighting the need of vessels to support cells in such large structures. To further understand this loss of metabolism we assessed the cell number and viability of cells at day 14, by decrosslinking the Alginate hydrogel and digesting cells with collagenase. Importantly, 78±9% compared to 21±11% cell viability in vascularized verses non-vascularized hydrogels was observed (*n*=3). Furthermore, we confirmed 5.7±2.4 × 10^7^ compared to 1.6±0.9 × 10^6^ viable cells/ml in vascularized and non-vascularized, respectively. As the starting cell density was 1 × 10^7^/ml this shows a ~5-fold expansion and ~6-fold loss of viable cell numbers, with and without vascularization, respectively. We therefore confirm viability and expansion of ihMSCs in these structures, with metabolism decreasing likely to be a function of cell density and proliferation/differentiation status.

## 4 Discussion

Most previous studies aiming to produce engineered tissue-like vasculature have employed layer-by-layer stacking of individually fabricated sheets. A technological improvement for the fabrication of such structures was achieved by employing 3D printed carbohydrate-dextran lattices as a sacrificial material to allow entire networks to be monolithically cast inside matrices to create tissue-like vessels in one step [5]. Building on this approach, we were able to create a temperature-sensitive, inexpensive sugar-based material that could be used in additive manufacturing and casting techniques to create stable and well-defined vascular networks theoretically for any 3D printable design and at human sizes. The developed GSM material has been tailored for its ease of application and flowability making it ideal for these applications. GSM can also be sterilized in pure chloroform further facilitating translation of its use. A denser material would be less flowable but create a more rigid material and allow higher resolution and accuracy. A less dense material would be easy to manipulate and flowable but not allow fine detail, intricate or accurate patterning. The formulation of GSM allows sufficient density and flowability to achieve both aims. We have demonstrated that GSM can be used to pre-fabricate vascular patterns (by moulding) or directly fabricate vascular patterns on prefabricated materials (PCL) without compromising biocompatibility and can be used with a wide variety of cells and tissue/matrix applications. Ideally such systems would support the process of anastomosis (cross connectivity by neovasularization) during tissue development post-fabrication. We believe that our 3D printed vascular constructs would support hydrogel remodelling by loaded cells (such as HUVECs) and differentiation using the suitable angiogenic factors (such VEGF). Employing hydrogels such as GelMA or fibrin could create the optimal mechanical properties sufficient for anastomosis and facilitate long-term continuous perfusion of already established networks [35].

Our material has significant benefits over using the previously described carbohydrate-dextran glass technologies [5]. GSM can be used on conventional 3D printers and can use PCL as a mould or scaffold during manufacturing to generate more mechanically strong constructs which can be used with slower crosslinking hydrogels. GSM does not confer mechanical properties to the resultant scaffolds, which is a function of the hydrogels and other materials. Also scaffold mechanics is linked to the development of tissue from progressive cell proliferation and ECM deposition. The material properties can be fine-tuned with simple change of humidity or temperature, converting a dry brittle material to an elastic, mouldable putty. No complex fabrication is required as for the ‘drawing’-based printing process used for carbohydrate-dextran glass [5]; this is because PCL is thermally extruded and GSM can then be either extruded or cast within the PCL structure. This attribute allows complex STL files (converted from patient CT scans) to be directly printed. We demonstrated this by printing vasculature within a human nose designed from a CT scan (Fig. 4). Importantly, GSM is temperature-sensitive meaning that PLGA-coating (as for carbohydrate-dextran glass) is not required to improve mechanical integrity during matrix casting. GSM is simply flowed out of the vessels post-matrix crosslinking by increasing the ambient temperature >37˚C; importantly this allows slow setting hydrogels such as collagen type-I and Matrigel™ to be used with the system. Unlike layer-by-layer processes, engineered constructs are vascularized in minutes and can be perfused directly post-evacuation of the GSM. Further modifications of GSM could allow more control over vessel circularity and surface smoothness and making GSM completely self-supporting without gelation. However, the attributes demonstrated allow precise control of vessel diameter and unlimited 3D architectural fabrication, meaning this technique is valuable in biofabrication efforts. Our material may now be used to aid human-scale tissue engineering efforts and create transplantable materials with significant vascular transport to prevent tissue necrosis and promote native physiological function.

## Author Contributions

J.E.D conceived and initiated the project. J.E.D, F. E. A, L. R-C, Z. S and H. M. E designed and performed experiments. J.E.D and J.Y supervised the project. J.E.D and H. M. E wrote the paper.

## Declaration of Competing Interest

The authors declare no competing financial interests.

## Appendix Supplementary materials

Image, application 1

Image, application 2
