# Supplementary material for: Human-scale tissues with patterned vascular networks by additive manufacturing of sacrificial sugar-protein composites
Source: Acta Biomater. 2020 Sep 1;113:339–49. doi: 10.1016/j.actbio.2020.06.012 (PMC7435351; doi:10.1016/j.actbio.2020.06.012)
Supplement: Supplementary file 1 [file mmc1.docx]

**Supplementary Figures**

**Figure S1. Cytocompatibility of ESM, GSM and BaCl_2_ Alginate crosslinkers.**

**Figure S1.** Cytocompatibility of ESM, GSM and BaCl_2_ Alginate crosslinkers. **A)** Viability of iHMSCs treated with 0-50% (w/v) of ESM or GSM for 12h and metabolic activity assessed by PrestoBlue^®^ assay. **B)** Viability of iHMSCs treated with BaCl_2_ crosslinker (0-1M) for 4h used in the vessel formulation to crosslink alginate during encapsulation of the sacrificial structure. n=3.

**Figure S2. ESM and GSM are compatible with optical techniques.**

**Figure S2.** ESM and GSM are compatible with optical techniques. Optical extinction for a 100µl sample of ESM formulation containing egg white protein (EWP), BSM containing Bovine serum albumin (BSA) or GSM containing Gelatin, compared to carbohydrate glass previously described ^[^[^1^](#_ENREF_1)^]^ at 25°C . Gelatin-containing samples were also tested at 37°C in which the gelatin constituent is liquid. Wavelengths were tested which are commonly used during biocompatible imaging and photopolymerization (365–550 nm).

**
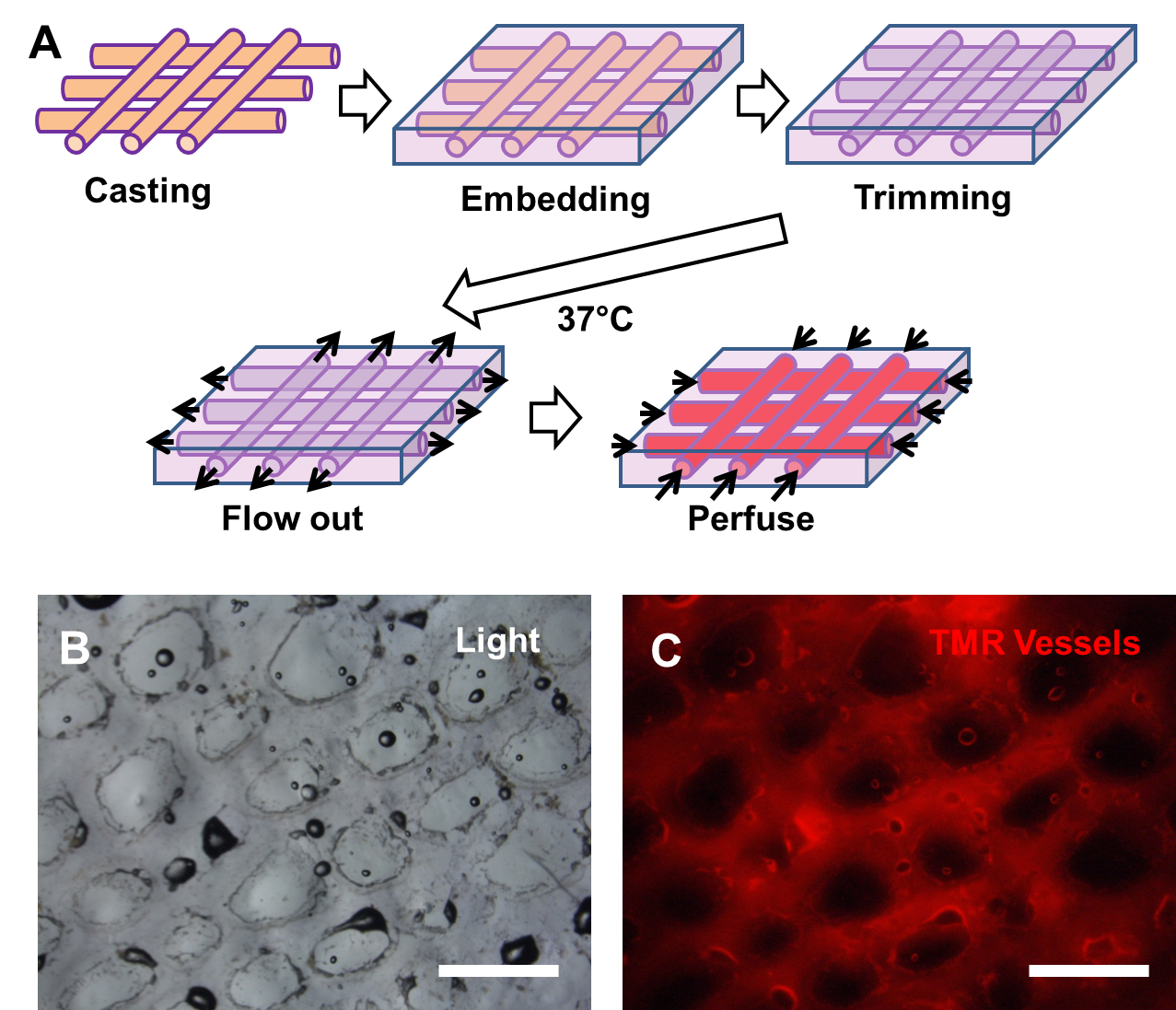
Figure S3. GSM-mediated vascularization forms faithful interconnected vessels.**

**Figure S3.** GSM-mediated vascularization forms faithful interconnected vessels. **A)** Schematic of the construction of vascularised constructs. Initially GSM is cast, moulded or printed, after the drying process the structure is embedded in hydrogel and the vessels opened by trimming of the hydrogel to allow the liquefied GSM (at 37°C) to flow out leaving open vessels that can be perfused. **B)** The vessel network can be visualised fluorescently by perfusing TMR (rhodamine) through the construct. Bar is 1mm.


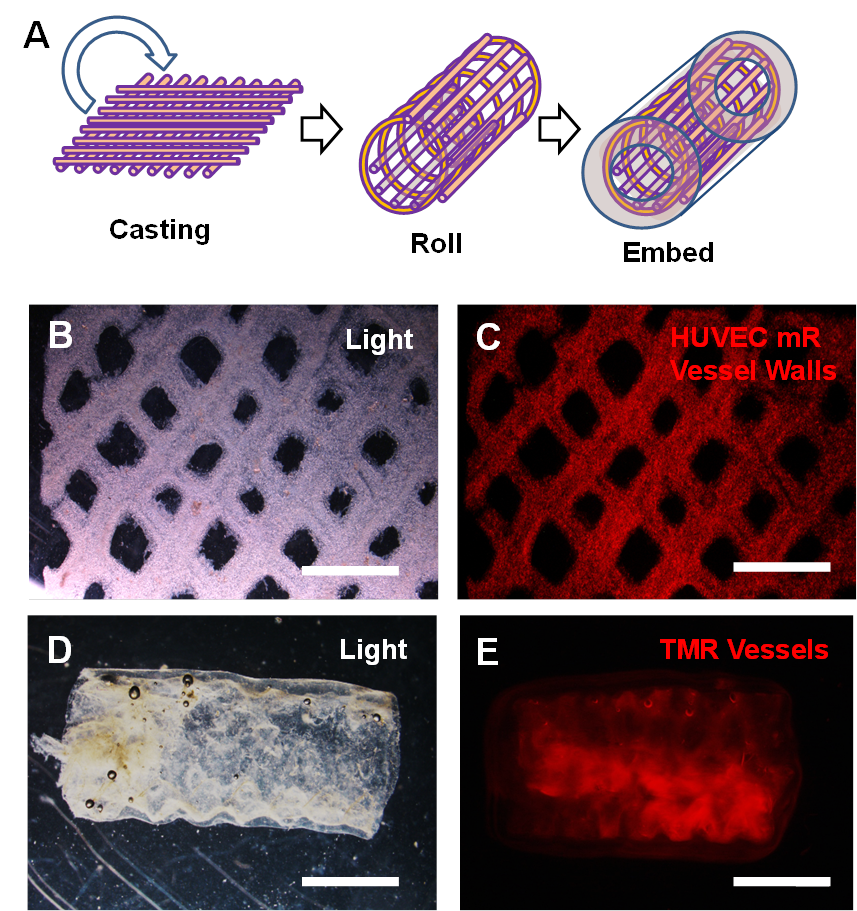
**Figure S4. GSM-mediated vascularization can be used directly to cellularize vessel walls and fabricated to produce larger vasculature constructs by rolling.**

**Figure S4.** GSM-mediated vascularization can be used directly to cellularize vessel walls and fabricated to produce larger vasculature constructs by rolling. **A)** Schematic of the construction of vascularized constructs. Initially GSM is cast, moulded or printed, post-setting the material can be rolled or stacked to form larger structures before embedding in hydrogel and the sacrificial material is removed. **B-C)** The vessel network walls can be cellularized by incorporating the hydrogel crosslinker into the GSM structure (BaCl_2_ and Alginate containing HUVEC-mR endothelial cells). Bar is 1mm. **D-E)** Larger vascularized structures such as tubes can be visualized fluorescently by perfusing TMR (rhodamine) through the construct. Bar is 3mm.

**Figure S5. GSM-mediated vascular networks can be used fabricated by PCL 3D printing and casting of GSM to produce thick multilayer vascularised constructs.**

**Figure S5.** GSM-mediated vascular networks can be used fabricated by PCL 3D printing and casting of GSM to produce thick multilayer vascularized constructs. **A)** Schematic of the construction of vascularized constructs. PCL is 3D printed layer-by-layer, after each layer GSM is cast, moulded or printed within the PCL layer building a composite multilayer construct. **B-C)** The composite GSM /PCL construct can be visualized by µCT both C) pre- and D) post-removal of PCL by chloroform extraction. Sacrificial GSM structure remains intact and the PCL is completely removed (example is 6 x 10 x 10 mm dimensions). Bars are 1mm and 300µm.

**Figure S6. GSM-mediated vascular networks can be used fabricated by injection moulding of wax 3D printed constructs produce thick multilayer vascularized constructs**

**
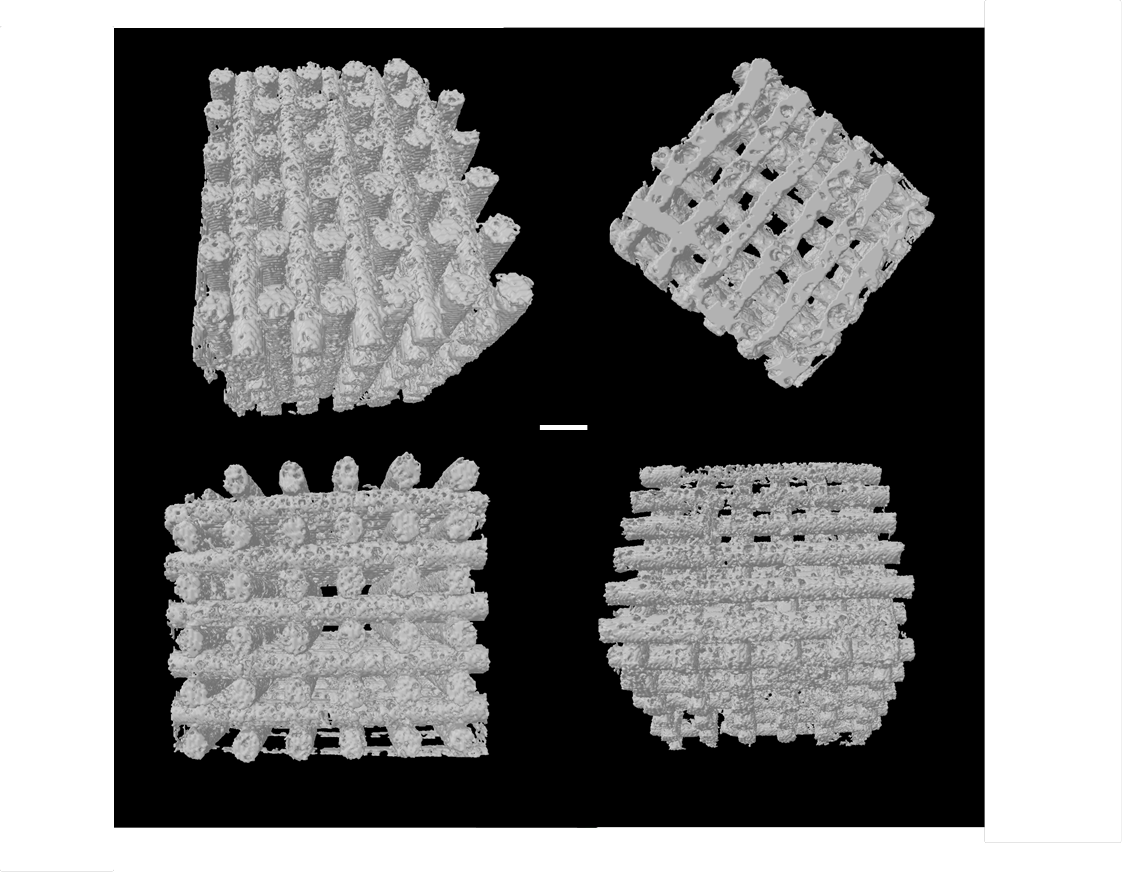
**

**Figure S6.** GSM-mediated vascular networks can be used fabricated by injection moulding of wax 3D printed constructs produce thick multilayer vascularized constructs. The wax mould (printed on a commercial 3D printer (ProJet^TM^ CPX 3000 plus; 3DSYSTEMS) can be used to mould GSM vessels and after wax removal with chloroform extraction can be visualized by µCT. Bar is 1mm.

**Figure S7. GSM vascularization is cell compatible and can be used with a variety of hydrogel systems which generate vessels with diffusible walls**.

**
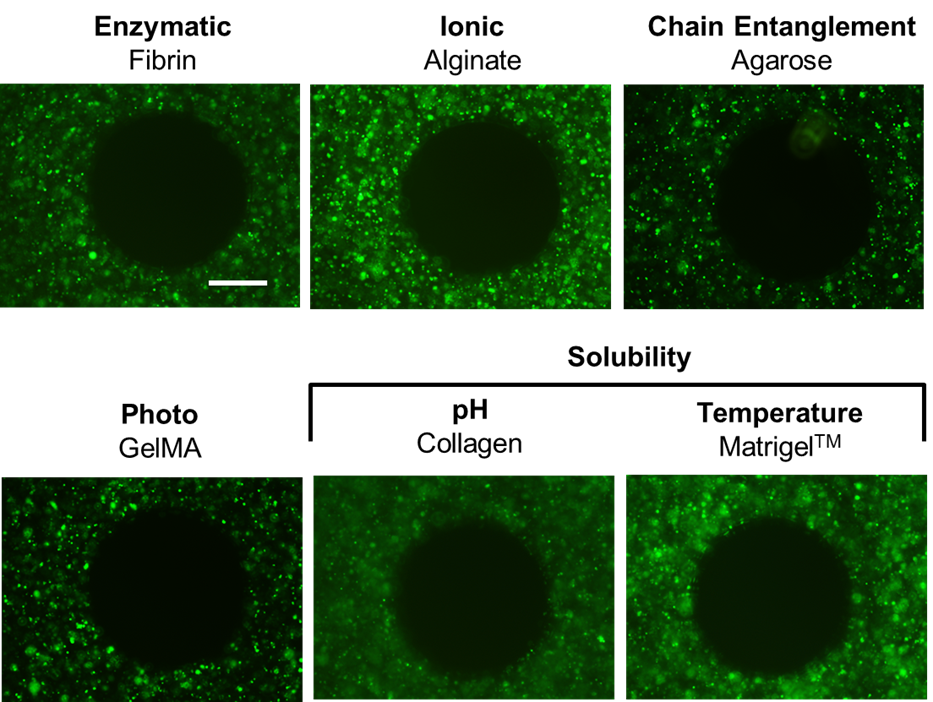
**

**Figure S7.** GSM vascularization is cell compatible and can be used with a variety of hydrogel systems. Mesenchymal stem cells (iHMSC-GFP) constitutively expressing enhanced green fluorescent protein (eGFP) were encapsulated (5x10^6^/ml) in a variety of hydrogel and ECM materials and then imaged with confocal microscopy to visualize the cells and the perfusable vascular lumen. The materials have varied crosslinking mechanisms (annotated above the images) but were all able to be patterned with vascular channels. Bar is 200 µm.

**Figure S8. GSM-mediated vascularization is biocompatible.**

**Figure S8.** GSM-mediated vascularization is biocompatible. We assessed the direct effect of GSM and its dissolution on cells encapsulated in Alginate (2% w/v) with and without GSM vascular structures. Cells were recovered 1 hour post-casting using de-crosslinking methods from [[2](#_ENREF_2)]). Viability was assessed by trypan-exclusion assay. We analysed immortalized human mesenchymal stem cells (iHMSCs), human induced pluripotent stem cells (HiPSCs), embryonic stem cells (HESCs), primary HMSCs and umbilical vein endothelial cells (HUVECs) which can all tolerate the process with no effect on viability. Bars are SD (n=3).


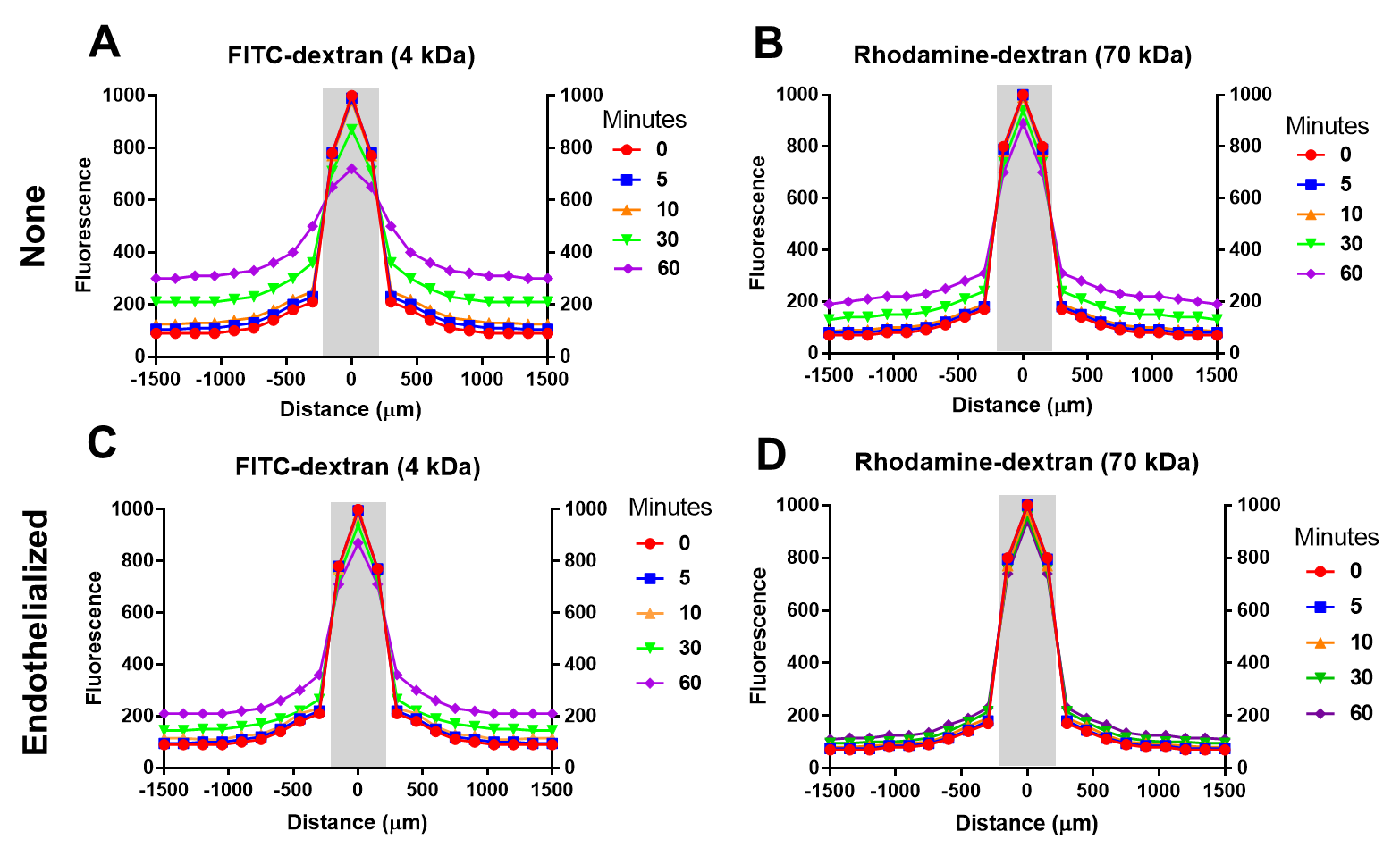
**Figure S9. Endothelialization of vessels assessed by diffusion**.

**Figure S9.** Endothelialization of vessels assessed by diffusion. Agarose gels with channels with diffusive transport of fluorescent dextran molecules, **A)** FITC-dextran, 4kDa or **B)** Rhodamine-dextran, 70kDa injected into the channel network. Agarose gels were also endothelialized with HUVECs in alginate before agarose casing, and **C)** FITC-dextran, 4kDa or **D)** Rhodamine-dextran, 70kDa diffusion assessed. Normalized fluorescence across the gel shows a sinusoidal profile in the vessel (grey hatched area) characteristic of a cylinder and temporal diffusion from the vessel into the bulk gel over time (0-60 mins). Vessels that were endothelialized were significantly reduced in diffusion levels and rates from the vessel into the bulk gel.

**Supplementary References:**

[1] J. S. Miller, K. R. Stevens, M. T. Yang, B. M. Baker, D. H. Nguyen, D. M. Cohen, E. Toro, A. A. Chen, P. A. Galie, X. Yu, R. Chaturvedi, S. N. Bhatia, C. S. Chen, Nature materials 2012, 11, 768.

[2] J. E. Dixon, D. A. Shah, C. Rogers, S. Hall, N. Weston, C. D. Parmenter, D. McNally, C. Denning, K. M. Shakesheff, Proceedings of the National Academy of Sciences of the United States of America 2014, 111, 5580.
